# A validated workflow for drug detection in oral fluid by non-targeted liquid chromatography-tandem mass spectrometry

**DOI:** 10.1007/s00216-018-1504-x

**Published:** 2018-12-06

**Authors:** Vera Reinstadler, Stefan Lierheimer, Michael Boettcher, Herbert Oberacher

**Affiliations:** 1 Institute of Legal Medicine and Core Facility Metabolomics, Medical University of Innsbruck, Muellerstr. 44, 6020 Innsbruck, Austria; 2 MVZ Labor Dessau GmbH, Bauhüttenstr. 6, 06847 Dessau-Roßlau, Germany

**Keywords:** Oral fluid, Saliva, Systematic toxicological analysis, Non-targeted analysis, Liquid chromatography-tandem mass spectrometry, Synthetic opioid

## Abstract

Oral fluid is recognized as an important specimen for drug testing. Common applications are monitoring in substance abuse treatment programs, therapeutic drug monitoring, pain management, workplace drug testing, clinical toxicology, and driving under the influence of drugs (DRUID). In this study, we demonstrate that non-targeted LC-MS/MS with subsequent compound identification by tandem mass spectral library search is a valuable tool for comprehensive detection and confirmation of drugs in oral fluid samples. The workflow developed involves solid-phase extraction and chromatographic separation on reversed phase materials. Mass spectrometric detection is accomplished on a quadrupole–quadrupole-time-of-flight instrument operated with data-dependent acquisition control. The workflow was optimized for 500 μl of neat oral fluid collected with the Greiner Bio-One saliva collection system. The fitness of the developed method was tested and proven by analyzing blank and spiked samples as well as 59 authentic patient samples. We could demonstrate that compounds with logP values in the range 0.5–5.5 are efficiently detected at low nanograms per milliliter concentrations. The true positive and true negative rates of automated library search were equal or close to 100%. The beauty of the non-targeted LC-MS/MS approach is the ability to detect compounds hardly included in routinely applied targeted assays, and this was demonstrated by detecting the synthetic opioid U-47700 in two patient samples.

**Electronic supplementary material:**

The online version of this article (10.1007/s00216-018-1504-x) contains supplementary material, which is available to authorized users.

**Graphical abstract:**
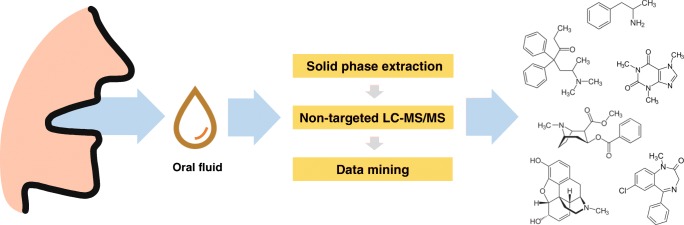
ᅟ

## Introduction

Oral fluid is recognized as an important specimen for drug testing [1]. Common applications are monitoring in substance abuse treatment programs, therapeutic drug monitoring, pain management, workplace drug testing, clinical toxicology, and driving under the influence of drugs (DRUID). Oral fluid can be used to confirm the recent consume of all major types of abused drugs [2–4]. Even new psychoactive substances (NPS) are detectable [5–8]. Furthermore, the monitoring of legally prescribed drugs such as benzodiazepines, z-hypnotics, methadone, and buprenorphine is common [9–11].

Advantages of using oral fluid include rapid, simple, and non-invasive collection, no requirement for medical personnel, and no gender collection issues. Furthermore, observed sampling effectively excludes the possibility of substitution and adulteration. These sampling characteristics render oral fluid drug testing a valuable and popular alternative to blood and urine testing.

Despite considerable success of oral fluid drug testing, for examination of the physiological effect of a consumed drug, blood testing is the golden standard. There is some correlation between blood and oral fluid concentrations suggesting that oral fluid is a useful substitute for blood [12–15]. However, attempts to establish fixed conversion factors failed for most drugs, due to large individual differences related to characteristics of the subjects such as age, gender, health, medication, sport, and nutrition.

Most drugs appear to enter saliva from blood by simple passive diffusion. Generally, basic and non-polar molecules having the properties of low protein binding and low molecular weight are ideal candidates for salivary monitoring. Thus, compounds, such as amphetamines, opiates, and cocaine, are often detected in higher concentrations in oral fluid than in plasma [16]. Others (e.g., benzodiazepines and 11-nor-9-carboxy-Δ9-tetrahydrocannabinol) are characterized by low saliva-to-plasma ratios [16, 17]. A clear challenge for drug testing of such compounds is achieving the necessary low limits of detection. Accordingly, laboratory analysis usually involves targeted analysis employing liquid chromatography-tandem mass spectrometry (LC-MS/MS) [2, 5–7, 9–11, 18–22]. Herein, we demonstrate the applicability of non-targeted LC-MS/MS for that purpose.

Non-targeted LC-MS/MS is an efficient method for comprehensive drug screening in various human specimens, including blood, urine, and hair [23, 24]. An integral step of non-targeted analysis is the generation of information-rich product ion mass spectra. Thus, any non-targeted LC-MS/MS technique involves detection of compounds eluting from the chromatographic column in MS and their submission to MS/MS. To accomplish automated selection and fragmentation, either “data-dependent acquisition” (DDA) or “data-independent acquisition” (DIA) techniques are employed. For compound identification, the acquired tandem mass spectral data is submitted directly or after deconvolution to tandem mass spectral library search.

In this study, we demonstrate that non-targeted LC-MS/MS in combination with tandem mass spectral library search represents a valuable tool for drug screening in oral fluid samples. The workflow was optimized for samples collected with the Greiner Bio-One saliva collection system (Greiner, Kremsmünster, Austria). It involves solid-phase extraction and chromatographic separation on reversed phase materials. Mass spectrometric detection is accomplished on a quadrupole–quadrupole-time-of-flight (QqTOF) instrument with DDA. The sample set that is used to evaluate and prove the fitness of the approach for the intended application consists of blank and spiked samples as well as 59 authentic patient samples that had been analyzed in a reference laboratory with a multitarget LC-MS/MS technique.

## Materials and methods

### Chemicals and samples

HPLC grade methanol (MeOH), formic acid (FA), acetic acid (HOAc), water, acetonitrile (ACN), sulfosalicylic acid, ethylene glycol, and ammonium acetate were purchased from Sigma-Aldrich (Schnelldorf, Germany).

The internal standards 2-ethylidene-1,5-dimethyl-3,3-diphenylpyrrolidine-D3, 3,4-methylenedioxyamphetamine-D5, 3,4-methylenedioxymethamphetamine-D5, 6-acetylmorphine-D6, amphetamine-D8, benzoylecgonine-D3, buprenorphine-D4, cocaine-D3, codeine-D6, methadone-D9, dihydrocodeine-D6, methamphetamine-D8, morphin-D3, and norbuprenorphine-D3 were obtained as solutions from Lipomed (Arlesheim, Switzerland) or Cerilliant (Round Rock, TX, USA).

Drug standards which were used for the preparation of spiked oral fluid samples were taken from the laboratory’s collection. The standards were either obtained from commercial suppliers (e.g., Lipomed or Cerilliant) or from the manufacturers of the marketed drugs.

Oral fluid was collected with the Greiner Bio-One saliva collection system (Greiner). The saliva content was determined with a photometric assay targeting the internal standard tartrazine. To enable the preparation of defined mixtures between oral fluids and the saliva extraction solution, expectorated oral fluids were used.

Anonymized oral fluid samples for routine drug screening were from opiate addicts in psychiatric hospitals (*N* = 10) or outpatients in maintenance therapy with either buprenorphine (*N* = 16), methadone (*N* = 28), or morphine (*N* = 5).

### Sample preparation workflow

**Fig. 1:**
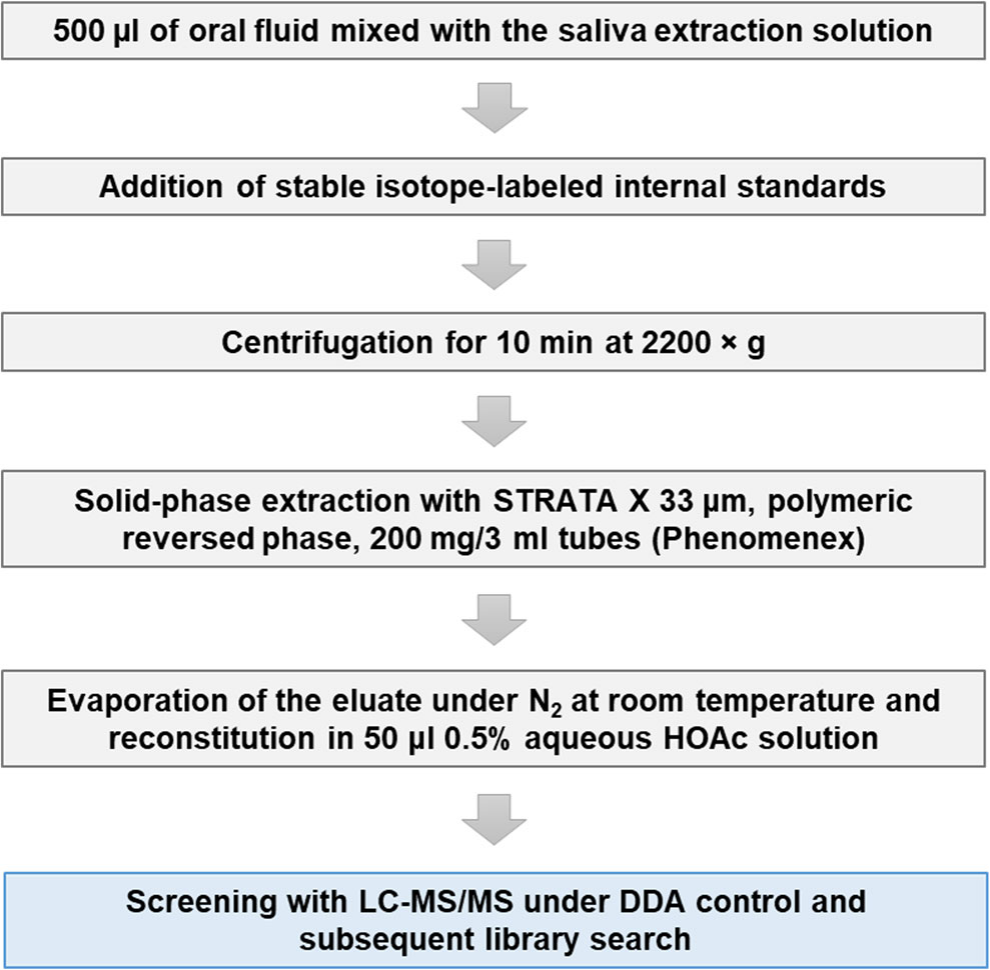
Overview on the principle steps of the sample preparation workflow employed for processing saliva samples collected with the Greiner Bio-One (GBO) saliva collection system

The principle steps of the sample preparation workflow are summarized in Fig. 1. An equivalent volume of 500 μl neat oral fluid was submitted to SPE after the addition of stable isotope-labeled internal standards (10 μl, buprenorphine-D4 and norbuprenorphine-D3 with 1000 ng/ml all other with 500 ng/ml) and centrifugation (2200×*g*, 10 min). SPE was accomplished on Strata-X cartridges (33 μm, 200 mg/3 ml, Phenomenex, Torrance, USA). SPE columns were washed with 2 ml MeOH and equilibrated with 2 ml water. Next, the sample was rinsed through the column. After washing with 3 ml water and 2 ml 30% MeOH in water (*v*/*v*), columns were dried under vacuum for 10 min to enable elution with two times 750 μl of 2% FA in ACN (*v*/*v*). The eluate was evaporated to dryness at 25 °C under a gentle stream of nitrogen. Finally, the dry residue was reconstituted in 50 μl aqueous 0.5% HOAc solution (*v*/*v*).

### Instrumentation

Samples were analyzed on an Eksigent 425 LC system hyphenated to a TripleTOF 5600+ (both Sciex, Framingham, MA, USA). Chromatographic separations were performed on a HALO Phenyl Hexyl column (150 × 0.5 mm, 2.7 μm, Sciex) by applying a linear gradient of 2–95% MeOH in aqueous 0.5% acetic acid solution (*v*/*v*) within 10 min. The column temperature was held at 50 °C. The flow rate was set to 15 μl/min. The Eksigent Expert 400 autosampler (Sciex) was used for sample injection. The injection volume was set to 5 μl. The mass spectrometer was operated in positive ESI mode using a DuoSpray ion source. The spray voltage was set to 5.5 kV. Gas flows of 40 arbitrary units for the nebulizer gas and 30 arbitrary units for the turbo gas were employed. The temperature of the turbo gas was adjusted to 200 °C. The instrument was operated at a mass resolution of ~ 30,000 for MS and ~ 15,000 for MS/MS, and automatically recalibrated every five sample injections using APCI positive calibration solution delivered via a calibration delivery system (Sciex). The scan range was *m/z* 100–700 for MS and *m/z* 50–700 for MS/MS. A duty cycle in the data-dependent acquisition mode included a single MS scan (accumulation time, 100 ms) followed by eight dependent MS/MS scans (accumulation time, 100 ms each) in the high sensitivity mode with dynamic background subtraction. The intensity threshold for triggering MS/MS experiments was set to 100 counts. MS/MS spectra were acquired at 35 eV with a collision energy spread of 10 eV. Former target ions were excluded for 30 s after two occurrences. The instrument was controlled by the Analyst TF 1.6 software (Sciex).

### Compound identification via automated library search

For data mining, we applied a recently described workflow that involves export of tandem mass spectral data, automated library search and expert reviewing [25–28].

Acquired fragment ion mass spectra were extracted from raw data files using MSConvert from ProteoWizard [29] and converted to plain text (ASCII) files with a program written in ActivePerl 5.6.1 (Active State Corporation, Vancouver, Canada). Compound identification was accomplished by tandem mass spectral library search using the “Wiley Registry of Tandem Mass Spectral Data, MSforID” (Wiley Registry MSMS) as reference library [30]. The Wiley Registry MSMS was developed on QqTOF instruments (TripleTOF 5600+, Qstar XL, both Sciex) [31, 32]. To cover the compound-specific breakdown curves by multiple reference spectra, for each compound, product ion mass spectra were acquired at ten different collision energy values ranging from 5 to 50 eV. Spectral curation included filtering of low abundant and unspecific signals [31, 33]. For this study, a library version was used that contained 20,377 spectra of 1709 entries. A detailed description of the library is provided on www.msforid.com.

Library search was accomplished with “MSforID Search”. A detailed description of the working principle can be found elsewhere [31, 34]. MSforID Search determines the similarities between the sample spectrum and each individual set of compound-specific reference spectra. This spectral comparison leads to two characteristic match probability values that may range between 0 and 100: (1) the “average match probability” (*amp*) and (2) the “relative average match probability” (*ramp*). High compound-specific match probability values indicate high similarity between the unknown spectrum and compound-specific reference spectra. The compound with the highest *amp* and *ramp* value, respectively, is considered to represent the unknown compound.

Automated MSforID Search was performed with a program written in Pascal using Delphi 6 for Windows (Borland Software Corporation, Scotts Valley, CA, USA; now Embarcadero Technologies, Inc., San Francisco, CA, USA) using the following search parameters: *m/z* tolerance of ± 0.01 and intensity cutoff factor of 0.01. A library search result was considered as putatively correct positive if the precursor ion mass error was within ± 0.01, the *amp* value > 5.0, and the *ramp* value > 40.0. The correctness of tentative identifications was checked by expert reviewing, which included visual inspection and comparison of tandem mass spectral data.

### Performance evaluation

Validation of the non-targeted LC-MS/MS workflow was accomplished as described previously for other specimens [25–28]. By analyzing blank samples, spiked samples, as well as authentic patient samples, the validation parameters selectivity, detection capability, and reliability of identification (sensitivity/specificity) were evaluated.

### Reference method

Authentic oral fluid samples were analyzed with a validated ultra-performance LC-MS/MS assay targeting 66 compounds (see Electronic Supplementary Material (ESM) Table S1). Fifty microliters of collected sample (mixture of oral fluid with extraction solution) was submitted to salting out liquid–liquid extraction after the addition of stable isotope-labeled internal standards (5 μl, 0.05–2.5 ng/ml each) and vortexing. After addition of 25 μl sulfosalicylic acid (20%), 50 μL ammonium acetate (10 M), and 350 μL ACN, the samples were vortexed and centrifuged. Three hundred twenty-five microliters of the supernatant was transferred and 10 μl ethylene glycol was added. After evaporation (N_2_, 45 °C), the samples were reconstituted with 10 μl MeOH and 80 μl water. An aliquot of 5 μL was injected into the LC-MS/MS system. The samples were measured on an Acquity UPLC I-Class system coupled with a Xevo TQ-S (Waters, Milford, MA, USA). Chromatographic separations were performed on an Acquity UPLC BEH Phenyl column (150 × 2.1 mm, 1.7 μm, Waters) by applying gradients of MeOH and ammonium formiate (20 mM) in aqueous 0.1% formic acid solution (*v*/*v*) within 5 min. The column temperature was held at 60 °C. The flow rate was set to 500 μl/min. Mass spectrometric detection was accomplished with ESI in positive mode employing multiple reaction monitoring.

## Results and discussion

### Overview on the principle steps involved in non-targeted LC-MS/MS of oral fluid samples

Non-targeted LC-MS/MS is a versatile technique for comprehensive drug testing [23, 24]. The technique is extensively used for analyzing blood, urine, and hair samples. Herein, we demonstrate that this concept is also useful for oral fluid analysis.

The workflow was optimized for the Greiner Bio-One saliva collection system. This device consists of the extraction solution (4 ml), a collection beaker with integrated transfer unit, and two vacuum transfer tubes (3.5 ml each) that contain stabilizers and preservatives. The extraction solution contains the yellow food dye tartrazine, which serves as internal standard enabling spectrophotometric quantification of the collected oral fluid volume. Arguments for choosing this device were the simple operation procedure, the possibility to accurately quantify the available oral fluid volume, and the already demonstrated excellent stability and recovery values [35, 36].

Although in this study only the Greiner Bio-One saliva collection system was used for oral fluid collection, principally, any common device should provide samples suitable for non-targeted LC-MS/MS analysis.

The oral fluid volume typically collected with the Greiner device ranges between 500 μl and 3 ml. Accordingly, 500 μl was defined as the oral fluid volume submitted to sample processing.

Stable-isotope-labeled analogues of 14 commonly observed drugs were selected as internal standards.

For isolation, cleanup, and preconcentration of the analytes of interest, a solid-phase extraction method employing polymeric reversed phase material with subsequent evaporation and reconstitution was used.

Chromatographic separations were accomplished on a phenyl hexyl column with a gradient of MeOH in aqueous 0.5% acetic acid solution. For mass spectrometric detection, a QqTOF instrument was employed.

DDA was selected as mode of operation for non-targeted LC-MS/MS. We are aware of the fact that DIA techniques would have allowed us to reach lower limits of detection [24]. However, as DIA would have significantly increased the time and effort spent for data mining, we decided to utilize DDA.

**Fig. 2:**
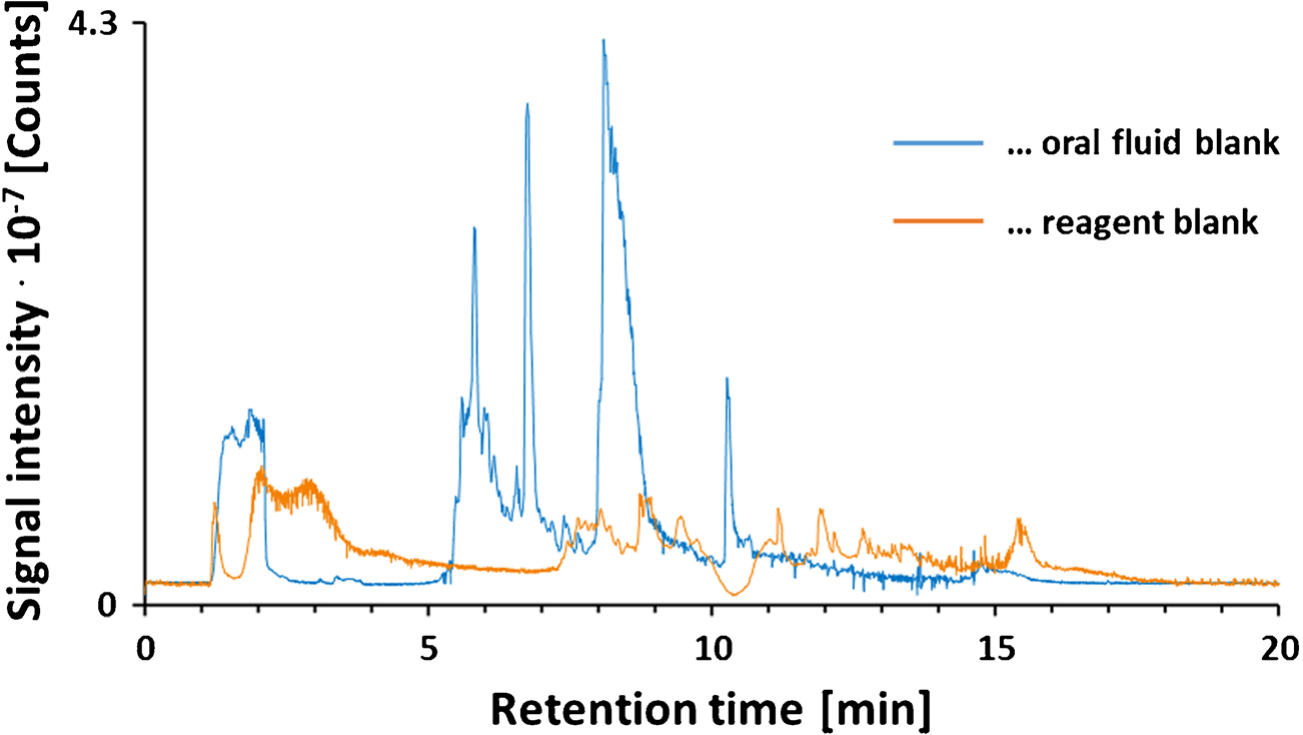
Total ion current chromatograms obtained from analyzing a blank and an oral fluid sample

Representative total ion current chromatograms (TICs) obtained from analyzing a reagent blank and an oral fluid blank are shown in Fig. 2. Five hundred microliters of water or oral fluid was mixed in the collection beaker with 400 μl of saliva extraction solution. Aliquots of the mixtures were transferred to vacuum transfer tubes and analyzed with the non-targeted LC-MS/MS workflow. Comparison of the obtained TICs clearly demonstrates that oral fluid introduces a significant load of matrix components. We are not able to provide any information on the identity of these compounds. However, as we were dealing with multiply charged ions, it is very likely that the detected biomolecules belong to the saliva peptidome.

Matrix can significantly hamper compound identification. It might induce ion suppression and can prevent fragmentation in DDA mode. To reduce the matrix load as much as possible during sample preparation, we decided to wash the SPE column prior to elution with 30% methanol. This washing step has a negative effect on the detectability of polar compounds (see below), but improves situation for other drug compounds.

The data mining strategy involved data export, tandem mass spectral library search, and expert reviewing of positive matches. Although a non-targeted data acquisition strategy was used, subsequent compound identification was a targeted process. This means that the number of identifiable compounds is limited to the targets included in the tandem mass spectral library selected. The library that we applied contained reference spectra of 1709 compounds.

### Evaluation of the performance of the LC-MS/MS method with blank and spiked oral fluid samples

Method validation is the process of sufficiently developing a picture of the performance of a method to demonstrate that it is fit for an intended purpose. To demonstrate the usefulness of the LC-MS/MS workflow for oral fluid drug testing, the following parameters were studied: selectivity, detection capability, and reliability of identification (sensitivity/specificity).

Selectivity of the identification workflow and specificity of automated library search were tested by analyzing five oral fluid samples donated from volunteers who did not consume any drug. Non-targeted LC-MS/MS produced 11,437 product ion mass spectra. By automated library search, 230 tentative positive identifications were obtained. Expert reviewing sorted out 83 false positive matches. Thus, specificity of library search was 99.3%. This low false positive rate is in agreement with previously published values for our data mining workflow [25, 26, 28]. The compounds correctly identified included nutritional compounds (e.g., caffeine, theophylline, and piperine), endogenous compounds (e.g., adenosine, tryptophan, phenylalanine, and cortisone), and some contaminants (e.g., diphenylamine, oxyquinoline, diphenylguanidine, and phtalimide). Of particular importance is the confirmation of endogenous compounds, which can be regarded a direct proof of matrix authenticity. As no identification of a drug compound occurred, the LC-MS/MS workflow clearly passed the selectivity test.

Detection capabilities of the developed LC-MS/MS method and the sensitivity of automated library search were tested by analyzing blank oral fluid samples fortified with 50 reference standards at seven different concentration levels (1.0 ng/mL, 2.5 ng/mL, 5.0 ng/mL, 10 ng/mL, 25 ng/mL, 50 ng/mL, and 100 ng/ml; see ESM Table S2).

**Fig. 3:**
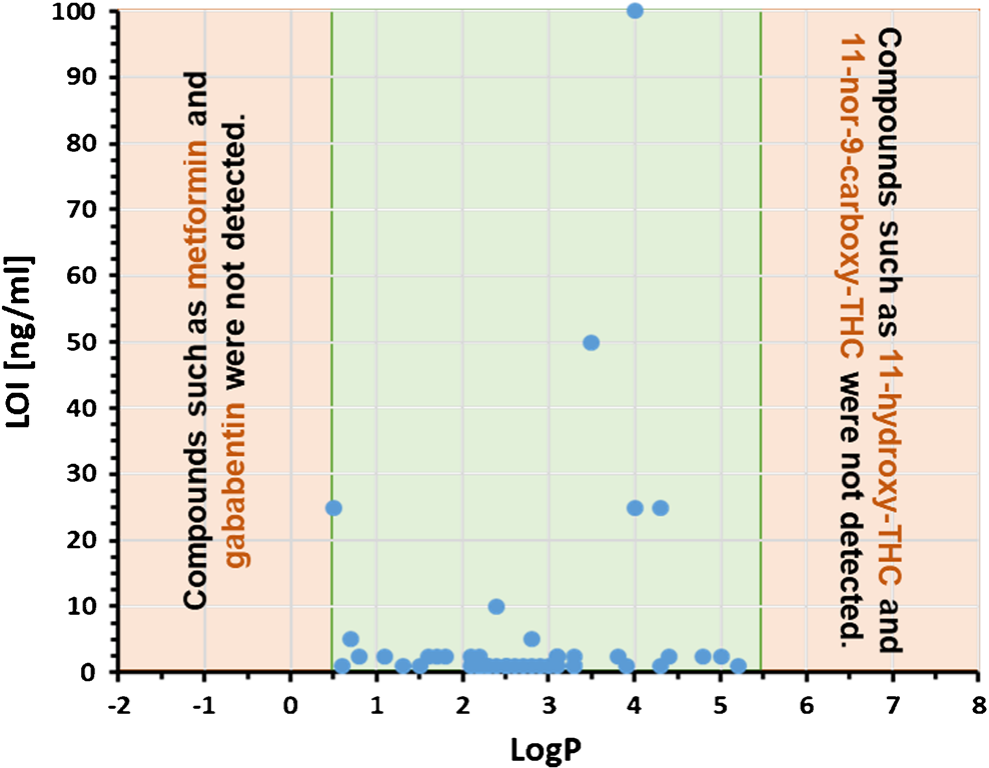
Dependence of the limits of identification (LOI) observed for 50 drug compounds from the corresponding logP values. The logP values were taken from PubChem (https://pubchem.ncbi.nlm.nih.gov/)

To enable the preparation of defined mixtures between oral fluids and the saliva extraction solution, expectorated oral fluids were used. Five hundred microliters of oral fluid was mixed with either 400 μl or 4 ml of saliva extraction solution. After spiking, the samples were analyzed and the minimum concentrations enabling identification of the compounds by automated library search were determined. Importantly, we observed no impact of the dilution factor on the detection capability. The majority of compounds (80%) showed limits of identification (LOI) of ≤ 5.0 ng/mL (Fig. 3). However, the workflow was found to have particular problems in detecting very polar (logP < 0.5; gabapentin, metformin, acetaminophen) and very apolar compounds (logP > 5.5; 11-hydroxy-Δ9-tetrahydrocannabinol, 11-nor-9-carboxy-Δ9-tetrahydrocannabinol). Polar compounds might either be lost in SPE or suppressed by coeluting species in LC-MS/MS. Apolar compounds got lost due to insufficient elution from SPE and chromatographic columns as well as limited solubility in the aqueous solvent used to reconstitute the evaporated SPE eluate.

Non-targeted LC-MS/MS workflows should be capable of detecting a broad range of chemicals. Our experiments clearly demonstrate that the applicability of any such method is restricted to a certain window of the entire chemical space. The window size is governed by the applied sample processing and chromatographic conditions. Our workflow works efficiently in the logP range 0.5 to 5.5. This enables the detection of almost all important illegal drug classes, except cannabinoids, with LOI values that are sufficiently low to fulfill the requirements for screening and confirmation methods issued by regulating authorities, including the Substance Abuse and Mental Health Services Administration (SAMSHA) and the European Workplace Drug Testing Society [37, 38].

For compounds within the logP range 0.5 to 5.5, LOI values beyond 10 ng/ml were only observed for fentanyl, ibuprofen, duloxetine, and fluoxetine. This observation might be attributable to either low ionization efficiencies or the production of unspecific fragmentation patterns. The later issue is of particular relevance for duloxetine and fluoxetine. These compounds decompose efficiently without producing abundant fragment ions in the applied scan range, which hampers identification of these compounds by library search. To overcome this problem, it would have been necessary to apply a collision energy of not more than 5–15 eV.

The tandem mass spectral data obtained from analyzing the spiked samples was further used to evaluate the sensitivity and specificity of automated library search. On average, 2060 tandem mass spectra were produced per run. The false positive rate was 1.6% only. The false negative rate was 0%. Obviously, if a compound-specific tandem mass spectrum is produced then the tandem mass spectral library will provide a correct match. A sensitivity value of 100% is in good agreement with values previously published for our database [25, 26, 28].

### Analysis of authentic oral fluid patient samples

Fifty-nine authentic patient samples were analyzed with the non-targeted LC-MS/MS workflow. The samples were collected from opiate addicts in psychiatric hospitals (*N* = 10) or outpatients in maintenance therapy with either buprenorphine (*N* = 16), methadone (*N* = 28), or morphine (*N* = 5) for drug screening. Each sample was screened twice. The obtained results were crosschecked with information on drug content obtained from a targeted LC-MS/MS technique. The reference method provides quantitative information for 51 compounds with cutoff values in neat oral fluid as low as 0.1 ng/ml (see ESM Table S1).

As already outlined above, with non-targeted LC-MS/MS, a significant number of endogenous compounds, nutritional compounds as well as contaminants are identifiable. These identifications, however, were not considered in the following evaluation.

**Fig. 4:**
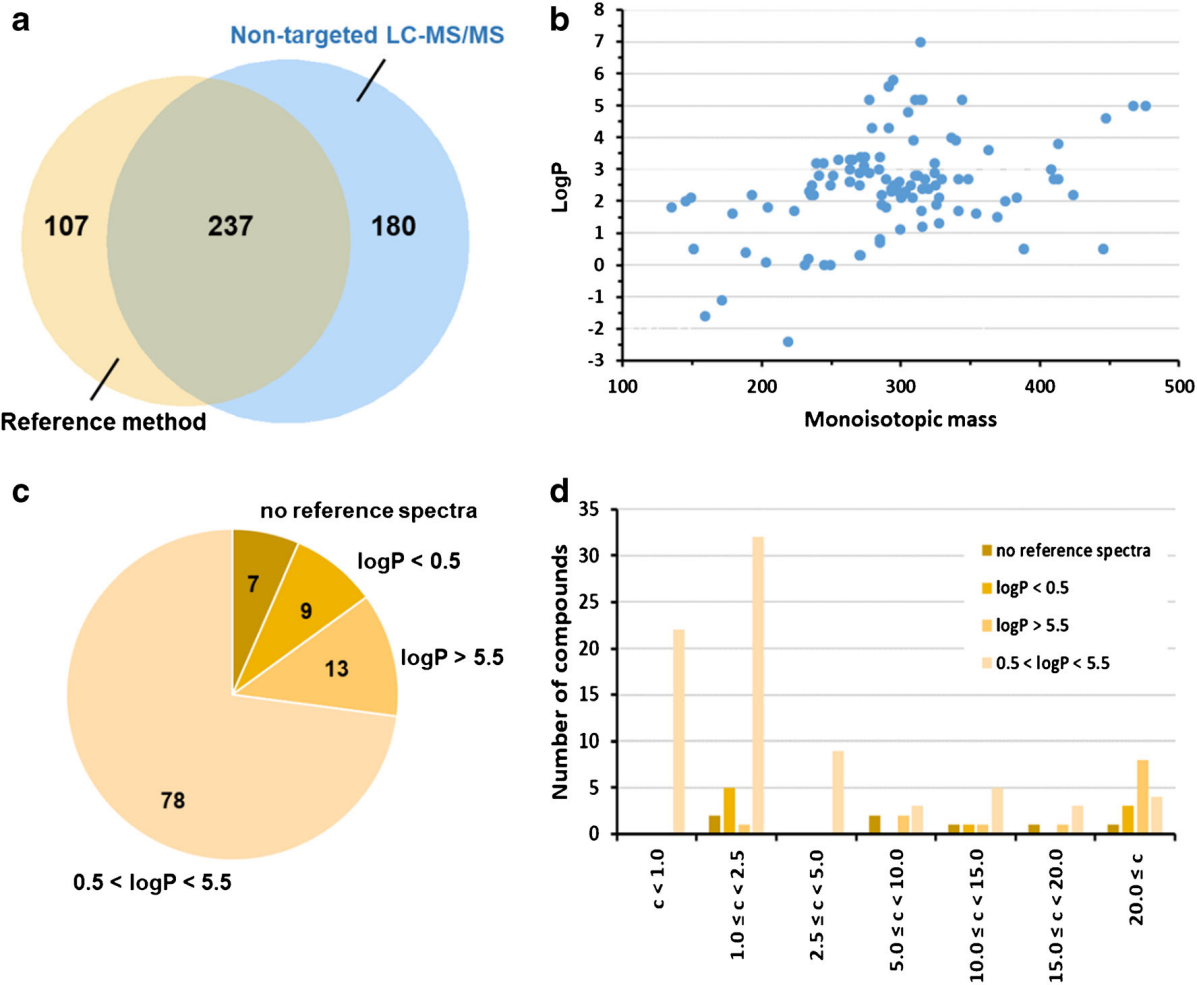
Validation of the non-targeted LC-MS/MS with a targeted reference method by analyzing 59 authentic patient samples. **a** Venn diagram showing the agreement between the analytical results. **b** Molecular characteristics of the 119 compounds identified by non-targeted LC-MS/MS. **c** Characterization of the 107 positive results obtained by the reference method only based on logP as well as the availability of reference spectra. **d** Characterization of 107 positive results obtained by the reference method only based on the measured oral fluid concentrations. The logP values were taken from PubChem (https://pubchem.ncbi.nlm.nih.gov/)

Comprehensive analysis of the patient samples with two different workflows led to 524 identifications of drug compounds or metabolites thereof (Fig. 4a). Two hundred thirty-seven identifications (45.2%) were obtained with both methods, 180 (34.4%) with non-targeted LC-MS/MS and 107 (20.4%) with the reference method only.

Four hundred seventeen identifications were obtained with the non-targeted LC-MS/MS workflow, and these represented 119 different compounds (Fig. 4b). The compounds spanned the entire chemical space accessible by this technique. Importantly, even a considerable number of very polar compounds (logP < 0.5) were detected. Obviously, in these cases, concentrations were sufficiently high to enable detection.

An overview on the 119 compounds identified is provided in ESM Table S3. Compounds with ten and more positive tests included methadone (*N* = 29), 2-ethylidene-1,5-dimethyl-3,3-diphenylpyrrolidine (EDDP, *N* = 25), diazepam (*N* = 16), buprenorphine (*N* = 14), nordazepam (*N* = 14), cocaine (*N* = 13), and morphine (*N* = 11). Positively tested illegal drugs included cocaine, delta(9)-tetrahydrocannabinol, 3,4-methylenedioxymethamphetamine, 3,4-methylenedioxyamphetamine, 6-acetylmorphine, and methamphetamine.

There were 180 cases of identifications that were only obtained with the non-targeted LC-MS/MS workflow (Fig. 4a). Overall, 78 different compounds were observed. These included 45 parent drugs and 33 drug metabolites. The majority of drugs detected represented antidepressants (e.g., doxepin, venlafaxine, trimipramine, mirtazapine, citalopram), antipsychotics (e.g., quetiapine, olanzapine, chlorprothixene, pipamperone, sertraline, sultopride), and analgesics (acetaminophen, antipyrine). This observation underlines the potential of oral fluid as clinical specimen for monitoring of psychopharmacotherapy [39].

An important observation was the detection of U-47700. U-47700 is a non-fentanyl synthetic opioid, which is clandestinely synthesized and distributed. U-47700 is primarily sold via the Internet. The compound is marketed as a heroin or an oxycodone substitute, as itself, or in combination with other drugs such as fentanyl. Since 2015, several confirmed fatalities associated with the presence of U-47700 in Europe and in the USA were reported. In these cases, U-47700 has been detected in blood and/or urine samples [40, 41]. We detected U-47700 together with its *N*-desmethyl metabolite in oral fluid and found 2 out of 59 samples being positively tested. This observation renders oral fluid testing with non-targeted LC-MS/MS a promising alternative to established surveillance tools for this and other synthetic opioids [8]. Furthermore, the prevalence of this designer opioid highlights the importance of comprehensive analysis for NPS, and this can only be accomplished with non-targeted LC-MS/MS.

We would like to emphasize that with extended compound coverage of the targeted method detection of those compounds that were only observed by non-targeted LC-MS/MS would have become possible. Usually, targeted LC-MS/MS methods are very reliable and provide low detection limits. Accordingly, only three out of the 180 cases of identifications that were solely obtained with the non-targeted LC-MS/MS workflow involved targets of the reference method. In one case, ketamine and, in two cases, temazepam were successfully detected. Evidence for correctness of the temazepam detections was provided by the observation of its parent drug diazepam as well as other diazepam metabolites (i.e., nordazepam, oxazepam) in the samples. There were 107 identifications that were obtained with the reference method only (Fig. 4a). In seven cases, reference spectra were missing in the database (Fig. 4c), rendering impossible the identification of these compounds (7-aminoclonazepam and nortilidine).

We tested the possibility of retrospective data analysis for the four samples containing 7-aminoclonazepam. After adding the corresponding set of reference spectra to the tandem mass spectral library, in three samples, the compound was correctly identified. In these cases, the concentration of 7-aminoclonazepam was found to be larger than 10 ng/ml. In the fourth sample, the 7-aminoclonazepam concentration was below 2 ng/ml, and thus the compound was not detected at all by non-targeted LC-MS/MS.

Missing reference spectra explained only a small number of the negative results obtained with the non-targeted LC-MS/MS method (6.5%). After removing the seven cases of 7-aminoclonazepam or nortilidine identifications, there were still 100 identifications left that were only obtained by the reference method. Next, we checked the raw data of the non-targeted LC-MS/MS runs for the availability of tandem mass spectral data representing the compounds missed. Importantly, none of the 100 cases such a tandem mass spectrum was available. This observation clearly indicates that data mining works efficiently. The false results seem to be caused by problems with compound detection.

Twenty-two negative results are explained by the presence of compounds with logP values outside the recommended window (0.5 < logP < 5.5, Fig. 4c). These drugs included ritalinic acid, pregabalin, gabapentin, and delta(9)-tetrahydrocannabinol.

Compound concentrations below or close to the individual LOI values might explain a further number of negative results. The reference method provides cutoff values in neat oral fluid as low as 0.1 ng/ml (see ESM Table S1). For non-targeted LC-MS/MS, the LOI values are typically in the range 1–5 ng/ml. Therefore, we expected that the majority of false results obtained with non-targeted LC-MS/MS would have been attributable to differences in the detection capabilities. In fact, in 63 false cases, the drug concentrations were found to be below 5.0 ng/ml, and in 22 cases even below 1.0 ng/ml (Fig. 4d).

For the 15 cases with no identification and drug concentrations beyond 5 ng/ml, the observed inability of non-targeted LC-MS/MS to produce tandem mass spectral information seems to be related to the working principle of DDA. That means that ions were not selected for MS/MS due to the facts that (1) they were not among the eight most abundant signals with an intensity higher than 100 counts and/or (2) they eluted within the 30 s exclusion window activated by a former target. Therefore, it is very likely that some of these compounds would have become detectable by applying DIA techniques [27].

## Conclusions

Non-targeted LC-MS/MS with DDA represent a valuable tool for comprehensive drug screening in oral fluid samples. By combining this technique with solid-phase extraction, LOI in the low nanograms per milliliter range are achieved. Accordingly, for all common drug compounds, the detection capabilities are sufficient to meet the requirements issued in the context of monitoring in substance abuse treatment programs, workplace drug testing, and DRUID. Targeted data mining involving tandem mass spectral library search enables reliable compound identification. Automated library search produced no false negative result and only a low number of false positive results (< 2%). However, the false positives are acceptable as they are sorted out by expert reviewing.

The strength of non-targeted LC-MS/MS is the ability to comprehensively detect and subsequently identify drug compounds in oral fluid. Per se, there are no restrictions. Consequently, in a representative set of 59 authentic patient samples, more identifications were obtained in comparison to a targeted workflow. As demonstrated with the detection of U-47700, this feature could be of particular advantage in the context of NPS analysis.

Limitations of the developed non-targeted LC-MS/MS workflow are related to chemical properties of drug compounds, the technique for automated acquisition of tandem mass spectral data, and the availability of reference spectra.

We have shown that due to the use of reversed phase techniques for sample preparation and chromatographic separation, usually only compounds with logP values in the range 0.5–5.5 are efficiently detected with LOI values in the low nanograms per milliliter range.

The working principle of DDA might also be responsible for false negative identifications when dealing with complex samples such as oral fluid that are characterized by a considerable load of abundant matrix signals.

Targeted data mining involves tandem mass spectral library search. Our database enables reliable identification. Sensitivity and specificity were found to be close to 100%. The database version used for this study contained 20,377 spectra of 1709 entries. This is a reasonably large number of compounds for drug screening applications. Nevertheless, as only a small part of the entire chemical space is covered, negative results cannot completely be excluded. As such, we need to intensify our efforts to increase the number of compounds included in our database as well as integrating third-party libraries.

After detailed evaluation of the strengths and weaknesses of our method, we came to the conclusion that it is fit for oral fluid drug testing within the above defined restrictions.

## Electronic supplementary material

ESM 1 (PDF 139 kb)

## References

[1] Huestis MA, Verstraete A, Kwong TC, Morland J, Vincent MJ, de la Torre R (2011). Oral fluid testing: promises and pitfalls. Clin Chem.

[2] Bosker WM, Huestis MA (2009). Oral fluid testing for drugs of abuse. Clin Chem.

[3] Drummer OH (2005). Review: pharmacokinetics of illicit drugs in oral fluid. Forensic Sci Int.

[4] Verstraete AG. Oral fluid testing for driving under the influence of drugs: history, recent progress and remaining challenges. Forensic Sci Int. 2005;150(2–3):143–50. 10.1016/j.forsciint.2004.11.023. 10.1016/j.forsciint.2004.11.023 15944054

[5] Kneisel S, Auwarter V, Kempf J (2013). Analysis of 30 synthetic cannabinoids in oral fluid using liquid chromatography-electrospray ionization tandem mass spectrometry. Drug Test Anal.

[6] de Castro A, Lendoiro E, Fernandez-Vega H, Steinmeyer S, Lopez-Rivadulla M, Cruz A (2014). Liquid chromatography tandem mass spectrometry determination of selected synthetic cathinones and two piperazines in oral fluid. Cross reactivity study with an on-site immunoassay device. J Chrom A.

[7] Strano-Rossi S, Anzillotti L, Castrignano E, Romolo F, Chiarotti M (2012). Ultra high performance liquid chromatography-electrospray ionization-tandem mass spectrometry screening method for direct analysis of designer drugs, “spice” and stimulants in oral fluid. J Chrom A.

[8] Griswold MK, Chai PR, Krotulski AJ, Friscia M, Chapman BP, Varma N, Boyer EW, Logan BK, Babu KM (2017). A novel oral fluid assay (LC-QTOF-MS) for the detection of fentanyl and clandestine opioids in oral fluid after reported heroin overdose. J Med Toxicol.

[9] Vindenes V, Yttredal B, Oiestad EL, Waal H, Bernard JP, Morland JG, Christophersen AS (2011). Oral fluid is a viable alternative for monitoring drug abuse: detection of drugs in oral fluid by liquid chromatography-tandem mass spectrometry and comparison to the results from urine samples from patients treated with methadone or buprenorphine. J Anal Toxicol.

[10] Kunkel F, Fey E, Borg D, Stripp R, Getto C (2015). Assessment of the use of oral fluid as a matrix for drug monitoring in patients undergoing treatment for opioid addiction. J Opioid Manag.

[11] Wagner E, Raabe F, Martin G, Winter C, Plorer D, Krause DL, et al. Concomitant drug abuse of opioid dependent patients in maintenance treatment detected with a multi-target screening of oral fluid. Am J Addict. 2018. 10.1111/ajad.12737. 10.1111/ajad.12737 29797622

[12] Vindenes V, Lund HME, Andresen W, Gjerde H, Ikdahl SE, Christophersen AS, Oiestad EL (2012). Detection of drugs of abuse in simultaneously collected oral fluid, urine and blood from Norwegian drug drivers. Forensic Sci Int.

[13] Wille SMR, Raes E, Lillsunde P, Gunnar T, Laloup M, Samyn N, Christophersen AS, Moeller MR, Hammer KP, Verstraete AG (2009). Relationship between oral fluid and blood concentrations of drugs of abuse in drivers suspected of driving under the influence of drugs. Ther Drug Monit.

[14] Toennes SW, Steinmeyer S, Maurer HJ, Moeller MR, Kauert GF (2005). Screening for drugs of abuse in oral fluid - correlation of analysis results with serum in forensic cases. J Anal Toxicol.

[15] Langel K, Gjerde H, Favretto D, Lillsunde P, Oiestad EL, Ferrara SD, Verstraete AG (2014). Comparison of drug concentrations between whole blood and oral fluid. Drug Test Anal.

[16] Gjerde H, Mordal J, Christophersen AS, Bramness JG, Morland J (2010). Comparison of drug concentrations in blood and oral fluid collected with the intercept sampling device. J Anal Toxicol.

[17] Lee D, Huestis MA (2014). Current knowledge on cannabinoids in oral fluid. Drug Test Anal.

[18] Huestis MA (2009). A new ultra performance-tandem mass spectrometry oral fluid assay for 29 illicit drugs and medications. Clin Chem.

[19] Di Rago M, Chu M, Rodda LN, Jenkins E, Kotsos A, Gerostamoulos D (2016). Ultra-rapid targeted analysis of 40 drugs of abuse in oral fluid by LC-MS/MS using carbon-13 isotopes of methamphetamine and MDMA to reduce detector saturation. Anal Bioanal Chem.

[20] Concheiro M, Gray TR, Shakleya DM, Huestis MA (2010). High-throughput simultaneous analysis of buprenorphine, methadone, cocaine, opiates, nicotine, and metabolites in oral fluid by liquid chromatography tandem mass spectrometry. Anal Bioanal Chem.

[21] Concheiro M, de Castro A, Quintela O, Cruz A, Lopez-Rivadulla M (2008). Determination of illicit and medicinal drugs and their metabolites in oral fluid and preserved oral fluid by liquid chromatography-tandem mass spectrometry. Anal Bioanal Chem.

[22] Lee PD, Chang YJ, Lin KL, Chang YZ (2012). Simultaneous determination of delta(9)-tetrahydrocannabinol and 11-nor-9-carboxy-delta(9)-tetrahydrocannabinol in oral fluid using isotope dilution liquid chromatography tandem mass spectrometry. Anal Bioanal Chem.

[23] Oberacher H, Arnhard K (2016). Current status of non-targeted liquid chromatography-tandem mass spectrometry in forensic toxicology. Trends Anal Chem.

[24] Oberacher H, Arnhard K (2015). Compound identification in forensic toxicological analysis with untargeted LC/MS-based techniques. Bioanalysis.

[25] Pitterl F, Kob S, Pitterle J, Steger J, Oberacher H (2016). Applying LC with low resolution MS/MS and subsequent library search for reliable compound identification in systematic toxicological analysis. LC GC Europe.

[26] Steger J, Arnhard K, Haslacher S, Geiger K, Singer K, Schlapp M, Pitterl F, Oberacher H (2016). Successful adaption of a forensic toxicological screening workflow employing nontargeted liquid chromatography-tandem mass spectrometry to water analysis. Electrophoresis.

[27] Arnhard K, Gottschall A, Pitterl F, Oberacher H (2015). Applying ‘sequential windowed acquisition of all theoretical fragment ion mass spectra’ (SWATH) for systematic toxicological analysis with liquid chromatography-high-resolution tandem mass spectrometry. Anal Bioanal Chem.

[28] Oberacher H, Schubert B, Libiseller K, Schweissgut A (2013). Detection and identification of drugs and toxicants in human body fluids by liquid chromatography-tandem mass spectrometry under data-dependent acquisition control and automated database search. Anal Chim Acta.

[29] Kessner D, Chambers M, Burke R, Agusand D, Mallick P (2008). ProteoWizard: open source software for rapid proteomics tools development. Bioinformatics.

[30] Oberacher H (2011). The Wiley Registry of Tandem Mass Spectral Data, MSforID.

[31] Pavlic M, Libiseller K, Oberacher H (2006). Combined use of ESI-QqTOF-MS and ESI-QqTOF-MS/MS with mass-spectral library search for qualitative analysis of drugs. Anal Bioanal Chem.

[32] Pavlic M, Schubert B, Libiseller K, Oberacher H (2010). Comprehensive identification of active compounds in tablets by flow-injection data-dependent tandem mass spectrometry combined with library search. Forensic Sci Int.

[33] Oberacher H, Pavlic M, Libiseller K, Schubert B, Sulyok M, Schuhmacher R, Csaszar E, Kofeler HC (2009). On the inter-instrument and inter-laboratory transferability of a tandem mass spectral reference library: 1. Results of an Austrian multicenter study. J Mass Spectrom.

[34] Oberacher H, Pavlic M, Libiseller K, Schubert B, Sulyok M, Schuhmacher R, Csaszar E, Kofeler HC (2009). On the inter-instrument and the inter-laboratory transferability of a tandem mass spectral reference library: 2. Optimization and characterization of the search algorithm. J Mass Spectrom.

[35] Langel K, Engblom C, Pehrsson A, Gunnar T, Ariniemi K, Lillsunde P (2008). Drug testing in oral fluid - evaluation of sample collection devices. J Anal Toxicol.

[36] Boettcher M, Peschel A (2011). Oral fluid levels of nicotine and metabolites in smokers as a function of collection device. Ther Drug Monit.

[37] Brcak M, Beck O, Bosch T, Carmichael D, Fucci N, George C, Piper M, Salomone A, Schielen W, Steinmeyer S, Taskinen S, Weinmann W (2018). European guidelines for workplace drug testing in oral fluid. Drug Test Anal.

[38] Flood JG, Khaliq T, Bishop KA, Griggs DA (2016). The new Substance Abuse and Mental Health Services Administration oral fluid cutoffs for cocaine and heroin-related analytes applied to an addiction medicine setting: important, unanticipated findings with LC-MS/MS. Clin Chem.

[39] Neumann J, Beck O, Dahmen N, Bottcher M (2018). Potential of oral fluid as a clinical specimen for compliance monitoring of psychopharmacotherapy. Ther Drug Monit.

[40] Mohr A, Friscia M, Papsun D, Kacinko S, Buzby D, Logan B (2016). Analysis of novel synthetic opioids U-47700, U-50488 and furanyl fentanyl by LC-MS/MS in postmortem casework. J Anal Toxicol.

[41] Fleming SW, Cooley JC, Johnson L, Frazee CC, Domanski K, Kleinschmidt K, Garg U (2017). Analysis of U-47700, a novel synthetic opioid, in human urine by LC-MS-MS and LC-QToF. J Anal Toxicol.

