# Supplementary material for: A validated workflow for drug detection in oral fluid by non-targeted liquid chromatography-tandem mass spectrometry
Source: Anal Bioanal Chem. 2018 Dec 6;411(4):867–76. doi: 10.1007/s00216-018-1504-x (PMC6338695; doi:10.1007/s00216-018-1504-x)
Supplement: Supplementary file 1 — (PDF 139 kb) [file 216_2018_1504_MOESM1_ESM.pdf]

**Analytical and Bioanalytical Chemistry**

**Electronic Supplementary Material**

# A validated workflow for drug detection in oral fluid by non-targeted liquid chromatography-tandem mass spectrometry

Vera Reinstadler, Stefan Lierheimer, Michael Boettcher, Herbert Oberacher

**Table S1** Compounds included in the targeted LC-MS/MS technique used to analyze 59 authentic patient samples

| Compound | Calibration range [ng/ml]* | Cutoff in neat oral fluid |
|---|---|---|
| Cortisol | 0.025-20.0 | 0.10 |
| Methadone | 0.025-20.0 | 1.0 |
| EDDP | 0.025-10.0 | 0.10 |
| Buprenorphine | 0.025-20.0 | 0.10 |
| Norbuprenorphine | 0.025-20.0 | 0.10 |
| Amphetamine | 0.025-20.0 | 5.0 |
| Methamphetamine | 0.025-20.0 | 5.0 |
| MDA | 0.025-20.0 | 1.0 |
| MDMA | 0.025-5.0 | 1.0 |
| MDEA | 0.025-5.0 | 1.0 |
| MBDB | 0.025-5.0 | 1.0 |
| BDB | 0.025-20.0 | 1.0 |
| PMMA | 0.025-5.0 | 1.0 |
| Methylone | 0.025-10.0 | 1.0 |
| Butylone | 0.025-10.0 | 1.0 |
| MDPV | 0.025-5.0 | 1.0 |
| Mephedrone | 0.025-2.5 | 1.0 |
| Pentedrone | 0.025-10.0 | 1.0 |
| 4-Methylethcathinone | 0.025-10.0 | 1.0 |
| alpha-PVP | 0.025-10.0 | 1.0 |
| Cocaine | 0.025-5.0 | 1.0 |
| Benzoylecgonine | 0.025-10.0 | 1.0 |
| Lidocaine | 0.025-1.5 | 1.0 |
| THC | 0.025-20.0 | 1.0 |
| Morphine | 0.025-20.0 | 1.0 |
| 6-Acetylmorphine | 0.025-20.0 | 1.0 |
| Noscapine | 0.025-5.0 | 0.10 |
| Codeine | 0.025-20.0 | 1.0 |
| Norcodeine | 0.025-20.0 | 1.0 |
| 6-Acetylcodeine | 0.025-10.0 | 1.0 |
| Dihydrocodeine | 0.025-20.0 | 1.0 |
| Tilidine | 0.025-2.5 | 1.0 |
| Nortilidine | 0.025-2.5 | 1.0 |
| Naloxone | 0.025-20.0 | 1.0 |
| Tramadol | 0.025-5.0 | 1.0 |
| O-Desmethyltramadol | 0.025-5.0 | 1.0 |
| Oxycodone | 0.025-20.0 | 1.0 |
| Noroxycodone | 0.025-20.0 | 1.0 |
| Hydromorphone | 0.025-20.0 | 1.0 |

| | | |
|---|---|---|
| Fentanyl | 0.025-2.5 | 0.10 |
| Dextromethorphan | 0.025-5.0 | 1.0 |
| Loperamide | 0.025-10.0 | 1.0 |
| Diazepam | 0.025-10.0 | 1.0 |
| Nordiazepam | 0.025-10.0 | 1.0 |
| Oxazepam | 0.025-10.0 | 1.0 |
| Temazepam | 0.025-10.0 | 1.0 |
| Flunitrazepam | 0.025-10.0 | 1.0 |
| 7-Aminoflunitrazepam | 0.025-10.0 | 0.10 |
| 7-Aminoclonazepam | 0.025-10.0 | 1.0 |
| Alprazolam | 0.025-10.0 | 1.0 |
| Bromazepam | 0.025-20.0 | 1.0 |
| Midazolam | 0.025-10.0 | 1.0 |
| Lorazepam | 0.025-10.0 | 1.0 |
| Flurazepam | 0.025-10.0 | 1.0 |
| Desalkylflurazepam | 0.025-5.0 | 1.0 |
| Phenazepam | 0.025-20.0 | 1.0 |
| Zaleplon | 0.025-5.0 | 1.0 |
| Zopiclone | 0.025-20.0 | 1.0 |
| Zolpidem | 0.025-2.5 | 1.0 |
| Methylphenidate | 0.025-2.5 | 1.0 |
| Ritalinic acid | 0.025-10.0 | 1.0 |
| Ketamine | 0.025-5.0 | 0.10 |
| Pregabalin | 0.025-20.0 | 2.0 |
| Gabapentin | 0.025-20.0 | 2.0 |
| Bupropion | 0.025-10.0 | 1.0 |
| Diphendydramine | 0.025-2.5 | 1.0 |

*Oral fluid mixed with an equal volume of the extraction solution was used for preparing calibration standards.

**Table S2** Evaluation of the detectability of 50 drugs in oral fluid with non-targeted LC-MS/MS under data-dependent acquisition control and subsequent library search by analyzing mixtures of these compounds in the concentration range 1.0-100 ng/ml

| Compound | Limit of identification [ng/ml] |
|---|---|
| 6-Acetylmorphine | 1.0 |
| Alprazolam | 1.0 |
| Benzoylecgonine | 1.0 |
| Cocaine | 1.0 |
| Diazepam | 1.0 |
| Dihydrocodeine | 1.0 |
| Doxepin | 1.0 |
| 2-Ethylidene-1,5-dimethyl-3,3-diphenylpyrrolidine | 1.0 |
| Heroin | 1.0 |
| Ketamine | 1.0 |
| Lidocaine | 1.0 |
| Methylenedioxypyrovalerone | 1.0 |
| Methadone | 1.0 |
| Methylone | 1.0 |
| Midazolam | 1.0 |
| Mirtazapine | 1.0 |
| Oxazepam | 1.0 |
| Temazepam | 1.0 |
| Tilidine | 1.0 |
| Tramadol | 1.0 |
| Trazodone | 1.0 |
| Triazolam | 1.0 |
| Venlafaxine | 1.0 |
| Zolpidem | 1.0 |
| Amphetamine | 2.5 |
| Bromazepam | 2.5 |
| Buprenorphine | 2.5 |
| Codeine | 2.5 |
| Diclofenac | 2.5 |
| Ketoprofen | 2.5 |
| 3,4-Methylenedioxyamphetamine | 2.5 |
| 3,4-Methylenedioxymethamphetamine | 2.5 |
| Methamphetamine | 2.5 |
| Morphine | 2.5 |
| Naproxen | 2.5 |
| Norbuprenorphine | 2.5 |
| Piroxicam | 2.5 |
| Sertraline | 2.5 |

| | |
|---|---|
| Mescaline | 5.0 |
| Olanzapine | 5.0 |
| Clonazepam | 10 |
| Acetaminophen | 25 |
| Duloxetine | 25 |
| Fentanyl | 25 |
| Ibuprofen | 25 |
| Fluoxetine | >100 |
| Gabapentin | >100 |
| Metformin | >100 |
| 11-Hydroxy-Δ9-tetrahydrocannabinol | >100 |
| 11-Nor-9-carboxy-Δ9-tetrahydrocannabinol | >100 |

**Table S3** Compounds identified in 59 authentic patient samples by the developed non-targeted LC-MS/MS workflow

| Compound | PubChem CID | Patients tested positive |
|---|---|---|
| 3,4-Methylenedioxyamphetamine | 1614 | 3 |
| 3,4-Methylenedioxymethamphetamine | 1615 | 5 |
| 6-Acetylmorphine | 5462507 | 5 |
| Acetaminophen | 1983 | 6 |
| Acetylcodeine | 5486550 | 3 |
| Alprazolam | 2118 | 1 |
| Amisulpride | 2159 | 1 |
| Amlodipine | 2162 | 1 |
| Amphetamine | 3007 | 5 |
| Antipyrine | 2206 | 5 |
| 4-Acetamidoantipyrine | 65743 | 5 |
| 4-Aminoantipyrine | 2151 | 1 |
| 4-Formylaminoantipyrine | 72666 | 4 |
| Aripiprazole | 60795 | 1 |
| Bisoprolol | 2405 | 4 |
| Bromazepam | 2441 | 3 |
| Hydroxy bromazepam | 166774 | 1 |
| Buprenorphine | 644073 | 14 |
| Norbuprenorphine | 114976 | 6 |
| Bupropion | 444 | 2 |
| Carbamazepine | 2554 | 1 |
| 10,11-trans-Dihydroxy-10,11-dihydrocarbamazepine | 83852 | 1 |
| Chlorpheniramine | 2725 | 1 |
| Chlorprothixene | 667467 | 2 |
| Citalopram | 2771 | 2 |
| N-Desmethyl citalopram | 162180 | 1 |
| Clindamycin | 446598 | 1 |
| Clobazam | 2789 | 1 |
| Norclobazam | 89657 | 1 |
| Clomipramine | 2801 | 1 |
| Clonazepam | 2802 | 3 |
| Cocaine | 446220 | 13 |
| Norcocaine | 644007 | 4 |
| Benzoylecgonine | 448223 | 8 |
| Cinnamoylcocaine | 5281863 | 2 |
| Cocaethylene | 644006 | 1 |
| Codeine | 5284371 | 5 |
| Norcodeine | 9925873 | 1 |
| delta(9)-Tetrahydrocannabinol | 16078 | 6 |

| | | |
|---|---|---|
| Dextromethorphan | 5360696 | 3 |
| Diazepam | 3016 | 16 |
| Nordazepam | 2997 | 14 |
| Oxazepam | 4616 | 6 |
| Temazepam | 5391 | 5 |
| Dihydrocodeine | 5284543 | 1 |
| Diphenhydramine | 3100 | 4 |
| N-Desmethyl diphenhydramine | 40791 | 3 |
| Doxepin | 667477 | 7 |
| Desmethyl doxepin | 6433351 | 7 |
| Doxylamine | 3162 | 3 |
| Eszopiclone | 969472 | 2 |
| Fentanyl | 3345 | 2 |
| Formoterol | 3410 | 1 |
| Gabapentine | 3446 | 1 |
| Hydroxy fentanyl | | 2 |
| Hydroxy mirtazapine | | 1 |
| Hydroxy quetiapine | | 3 |
| Hydroxy zolpidem | | 1 |
| Ketamine | 3821 | 3 |
| Norketamine | 123767 | 2 |
| Levamisole | 26879 | 1 |
| Lidocaine | 3676 | 2 |
| Loperamide | 3955 | 1 |
| Lorazepam | 3958 | 6 |
| Melperone | 15387 | 1 |
| Methadone | 4095 | 29 |
| 2-Ethylidene-1,5-dimethyl-3,3-diphenylpyrrolidine | 5378015 | 25 |
| Methamphetamine | 1206 | 5 |
| Methotrimeprazine | 26430 | 1 |
| Methylphenidate | 4158 | 4 |
| Ritalinic acid | 86863 | 5 |
| Metoclopramide | 4168 | 1 |
| Midazolam | 4192 | 1 |
| Hydroxy midazolam | 107917 | 1 |
| Mirtazapine | 4205 | 4 |
| N-Desmethyl mirtazapine | 10467350 | 4 |
| Morphine | 5288826 | 11 |
| Normorphine | 5462508 | 1 |
| Naloxone | 5284596 | 2 |
| Narceine | 8564 | 1 |
| Noscapine | 31411 | 4 |
| Olanzapine | 4585 | 3 |
| N-Desmethyl olanzapine | 9948206 | 2 |

| | | |
|---|---|---|
| Ondansetron | 4595 | 1 |
| Opipramol | 9417 | 1 |
| Oxycodone | 5284603 | 2 |
| Papaverine | 4680 | 4 |
| Pipamperone | 4830 | 2 |
| Pregabalin | 5486971 | 3 |
| Propylhexedrine | 7558 | 1 |
| Prothipendyl | 14670 | 1 |
| N-Desmethyl prothipendyl | | 1 |
| N-Didesmethyl prothipendyl | | 1 |
| Quetiapine | 5002 | 6 |
| N-Desalkyl quetiapine | 11369918 | 6 |
| O-Desalkyl quetiapine | | 3 |
| Quinine | 3034034 | 3 |
| Risperidone | 5073 | 1 |
| Sertraline | 68617 | 2 |
| Norsertraline | 114743 | 2 |
| Sulfapyridine | 5336 | 1 |
| Sultopride | 5357 | 2 |
| Terbinafine | 1549008 | 1 |
| Tilidine | 30131 | 4 |
| Torsemide | 41781 | 1 |
| Tramadol | 33741 | 3 |
| N-Disdesmethyl tramadol | 3056578 | 1 |
| Trimipramine | 5584 | 5 |
| Trimipramine N-Oxide | 14151360 | 2 |
| U-47700 | 13544016 | 2 |
| N-Desmethyl U-47700 | | 2 |
| Venlafaxine | 5656 | 7 |
| N-Desmethyl venlafaxine | 3501942 | 1 |
| O-Desmethyl venlafaxine | 125017 | 3 |
| N,N-Didesmethyl venlafaxine | 9795857 | 3 |
| N,O-Didesmethyl venlafaxine | | 1 |
| Venlafaxine N-Oxide | 76559643 | 1 |
| Xylometazoline | 5709 | 3 |
| Zolpidem | 5732 | 1 |
